# Impact of the vaginal applicator and dummy pellets on the dosimetry parameters of Cs‐137 brachytherapy source

**DOI:** 10.1120/jacmp.v12i3.3480

**Published:** 2011-05-19

**Authors:** Sedigheh Sina, Reza Faghihi, Ali S. Meigooni, Simin Mehdizadeh, M. Amin Mosleh Shirazi, Mehdi Zehtabian

**Affiliations:** ^1^ School of Mechanical Engineering Shiraz University Shiraz Iran; ^2^ Radiation Research Center Shiraz University Shiraz Iran; ^3^ Comprehensive Cancer Center of Nevada Las Vegas Nevada USA; ^4^ Radiotherapy Physics Unit Department of Radiology Shiraz University of Medical Sciences Shiraz Iran

**Keywords:** brachytherapy, dosimetry, Monte Carlo simulation, MCNP4C

## Abstract

In this study, dose rate distribution around a spherical ${}^{137}\text{Cs}$ pellet source, from a low‐dose‐rate (LDR) Selectron remote afterloading system used in gynecological brachytherapy, has been determined using experimental and Monte Carlo simulation techniques. Monte Carlo simulations were performed using MCNP4C code, for a single pellet source in water medium and Plexiglas, and measurements were performed in Plexiglas phantom material using LiF TLD chips. Absolute dose rate distribution and the dosimetric parameters, such as dose rate constant, radial dose functions, and anisotropy functions, were obtained for a single pellet source. In order to investigate the effect of the applicator and surrounding pellets on dosimetric parameters of the source, the simulations were repeated for six different arrangements with a single active source and five non‐active pellets inside central metallic tubing of a vaginal cylindrical applicator. In commercial treatment planning systems (TPS), the attenuation effects of the applicator and inactive spacers on total dose are neglected. The results indicate that this effect could lead to overestimation of the calculated F(r,θ), by up to 7% along the longitudinal axis of the applicator, especially beyond the applicator tip. According to the results obtained in this study, in a real situation in treatment of patients using cylindrical vaginal applicator and using several active pellets, there will be a large discrepancy between the result of superposition and Monte Carlo simulations.

PACS number: 87.53.Jw

## I. INTRODUCTION

The Selectron low‐dose‐rate (LDR) remote afterloading system distributed by Nucletron (Nucletron BV, Veenendaal, The Netherlands) is mainly used in gynecological brachytherapy to deliver the prescribed dose via different combinations of active spherical Cs‐137 pellet sources (supplied by Amersham Corporation, Louisville, CO) and inactive (dummy) pellets into applicator sets.^(1)^ Different combinations of active and inactive pellets are used for treatment of cervix and vaginal cancer. The Nucletron PLATO treatment planning system (TPS) calculates the dose delivered by the Selectron unit by treating the active pellets as point sources and by summing the dose contributions from the individual sources placed inside the applicator, at the point of interest.^(1)^ Dose calculation by simple superposition accounts only for the source filtration and does not take in to account the attenuation of photons by adjacent active or inactive pellets and by the applicator set. It is necessary to evaluate the applicator and inactive pellet attenuation effects on dose rate distributions. Markman et al.^(2)^ investigated the interapplicator attenuating effects for a typical intrauterine and double ovoid combination set by MC calculations, and showed that neglecting interapplicator shielding effects may decrease the accuracy of treatment. Siwek et al.^(3)^ showed the effect of applicator and other pellets on dose rate distributions on the tip of the applicator for a typical clinical configuration of pellets experimentally. According to their work, the dose is reduced up to 20% at the tip of applicator. Pérez Calatayud et al.^(4)^ calculated the dose distributions around a single active pellet without the applicator using Geant4 Monte Carlo (MC) code. They performed a complete Monte Carlo simulation for a typical train source configuration (Cs‐137 pellet sources, remote afterloading system plastic guide tube and gynecological applicator) and showed that applying the superposition principle to Monte Carlo data of the individual pellet sources would cause significant differences in dose estimations, especially towards the tip of the applicator. Therefore, it is very important to take the shielding effect of the applicator and other dummy pellets into account^(4)^ to reduce the errors and uncertainties in clinical treatment planning. The expertise and skill of the operator in optimal applicator insertion^(5)^ and the ability of the system in limiting the movement of the applicators are very important in gynecological brachytherapy, as the applicator displacement would worsen the local control and increase the morbidity.^(6)^

The purpose of this research is to investigate the dosimetry parameters of an individual low‐dose‐rate Cs‐137 Selectron pellet source within the 0.6 cm diameter central metallic tubing of a vaginal cylindrical applicator using Monte Carlo simulation and experimental techniques, following the recommendations of Task Group #43 (TG‐43) of the American Association of Physicists in Medicine (AAPM).^(^ ^7^ ^,^ ^8^ ^)^ In this study, the radial dose functions and anisotropy functions of a single pellet source in different positions inside the applicator were measured and calculated for different distances and angles in presence of the central metallic tubing of a vaginal applicator and inactive pellets. Having the TG‐43 parameters of the single pellet in different positions inside the applicator, we can determine the dose distributions around every configuration of active and inactive pellets by adding the dose from each pellet; there is no need to do a separate Monte Carlo simulation for every configurations used to treat the patients with vaginal cancer. These parameters will be utilized to update the data in the treatment planning systems in order to determine accurate radiation delivery for treatment of patients using such LDR units.

## II. MATERIALS AND METHODS

### A.1 Source geometry and composition

The Selectron remote afterloading sources consist of spherical Cs‐137 pellets, composed of 1.5 mm active source core of ceramic, encapsulated in 0.5 mm steel, with an overall diameter of 2.5 mm.^(9)^ In addition, this afterloading system contains some non‐active (dummy) sources, which have the same dimensions and chemical compositions as the active source. Different combinations of active and nonactive pellets are inserted into the applicator to deliver the prescribed dose in brachytherapy treatments.

### A.2 Dosimetry technique

In 1995, AAPM Task Group 43 (TG‐43) introduced a protocol for dosimetric evaluation of low‐energy brachytherapy sources used for interstitial brachytherapy.^(7)^ This protocol is used around the globe and provides a user‐friendly condition for clinical brachytherapy. In 2004, this protocol was further updated^(8)^ to remove the shortcomings that were in the original protocol.

In this protocol, the general 2D dose rate distribution around a brachytherapy source is defined as

(1) $$\dot{D}(r,\theta )=Sk\Lambda \frac{G_{x}(r,\theta )}{G_{x}(1cm,\pi /2)}g_{x}(r)F(r,\theta )$$

where $\overset{\cdot }{D}(r,\theta )$ is the dose rate at the point of interest at some distance *r* from the center of the source and polar angle θ relative to the longitudinal axis of the source, *Sk* is the air kerma strength, Λ is the dose rate constant in water, $G^{x}(r,\theta )$ is the geometry function at the point of interest, $G^{x}$ (1 cm,Π/2) is the geometry function at the reference point 1 cm from the source and at 90° from the longitudinal axis of the source, $g^{X}(r)$ is the radial dose function, and *F*(*r*,θ) is the 2D anisotropy function. The subscript *X* in the geometry and radial dose functions indicates the point and line source approximation in the geometry function. For a detailed description of the above parameters see the updated AAPM TG‐43 U1 protocol.

### A.3 Monte Carlo calculations

The MCNP4C code^(^ ^10^ ^,^ ^11^ ^)^ used in this study for determination of dosimetry parameters of Selectron Cs‐137 source is a general purpose Monte‐Carlo radiation transport code which is able to consider photoelectric, coherent, Compton and pair production interaction processes.

In the simulation geometry used to score dose rate constant and radial dose function of a single active pellet without the presence of applicator and other inactive pellets, the source was located at the center of a spherical water phantom with dimensions big enough (60 cm diameter) to provide the full scattering conditions. Because of the spherical symmetry of the source, there is no anisotropy around the source, so concentric spherical shells with 0.05 cm thickness were simulated around the source in order to calculate dose around the source at different distances from the source center. In order to investigate the effect of the applicator and inactive pellets on dosimetry parameters of the source, the simulations were done in a spherical water phantom with a single pellet source inside central metallic tubing of a vaginal applicator while other pellets are inactive. Tally cells used in this investigation were small spherical cells of 0.5 mm radius whose center is located at the calculation point and, in order to investigate the effect of the position of the single active pellet inside the applicator on dosimetry parameters, the simulations were done for an active pellet in six different positions inside the applicator.

**Figure 1:**
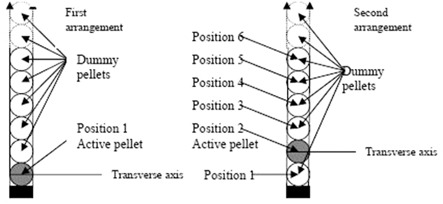
Geometry used in MCNP simulations. The simulations were done 6 times while a single pellet source was in positions 1 to 6.

TG‐43 dosimetric parameters of the source, such as dose rate constant, radial dose functions and anisotropy functions, were calculated with and without the applicator and dummy pellets in each arrangement. The geometry of the simulations is shown in Fig. 1. In the first arrangement, as shown in Fig. 1, the active pellet was considered to be in position 1, and all TG‐43 parameters were obtained. The simulations were repeated for five other arrangements in which the active pellet is in other positions (position 2 to position 6) and each time the dosimetry parameters were obtained to investigate the effect the position of the pellet on dose rate distribution of the source.

There are many different tally types available in MCNP4C for scoring diverse physical characteristics. Tally type F6 (energy deposition averaged over a volume Mev per gram per photon) was used to calculate dose rate distribution around the source, but as F6 is a kerma estimator, it is only valid when electronic equilibrium exists. Comparison of results of tally F6 with the results of tally *F8 (energy deposited in a cell) indicates that for 662 keV photon of Cs‐137 sources, the electronic equilibrium exists at distances greater than 2 mm from the source; so collision kerma closely approximates the absorbed dose and tally F6 can be used to score absorbed dose. Furthermore, we converted dose units of MeV/g per photon to Gy $h^{–1}U^{–1}$ by using the tally multiplier (FMn) card. In order to score the air kerma strength, Sk, as a measure of brachytherapy source strength, the air kerma rate was calculated at distances ranging from 0.5 to 150 cm by placing the tally cells in a 4 m diameter air volume, and by using the product of free‐space air kerma rate, and the distance d squared. The energy cutoff δ=10 keV was considered in this calculation. The densities and chemical composition of the applicator, and active and inactive pellets used in these simulations were obtained from the work of Pérez et al.;^(4)^ while the chemical composition and density of water and air phantoms were obtained from TG‐43U1 report.

The simulations for dose rate constant, radial dose function, and 2D anisotropy function were performed for up to 1.6×10 ^8^ starting particle histories in liquid water. However, for determination of dose rates on the longitudinal axis of the sources, the simulations were performed for up to 2×10 ^9^ starting particle histories.

From these simulations, the total errors at radial distances of 1.0 cm and 5.0 cm were found to be 0.3% and 1.5%, respectively.

### A.4 Thermoluminescence dosimetry

Dose rate distributions around the Cs‐137 pellet source were measured in homemade Plexiglas phantoms using Li‐F thermoluminescence dosimeter, TLD100 chips of dimensions 3×3×1 mm^3^. The chips were annealed using standard procedure.^(12)^ A delay of six days between the end of the irradiation and reading of the TLD chips was used to ensure the stabilization of the TLD. The irradiated TLDs were read using a Harshaw/Bicron Model 4500 TLD reader (Now Thermo Fisher Scientific Inc., Waltham, MA). The following equation was used to calculate the absorbed dose rate per air kerma strength from the TLD responses for each point irradiated in the phantom:^(^ ^12^ ^,^ ^13^ ^,^ ^14^ ^,^ ^15^ ^)^

(2) $$\frac{\dot{D}(r,\theta )}{Sk}=\frac{R}{T\;Sk\;\;\;\;\;E(r)d(T)Flin}$$

where $\overset{\cdot }{D}(r,\theta )$ is the initial absorbed dose rate at the start of the experiment at a point (r, θ), *R* is the TLD response corrected for background and the physical differences between the TLD chips using the predetermined chip factors,^(12)^ and *T* is the exposure time. In these measurements, the exposure time was selected such that the absorbed doses ranged from 10 to 100 cGy for which TLD responses were linear. *Sk* is the initial source air‐kerma strength at the start of the measurement. This parameter had been determined two months before the time of this investigation. We used this result by considering radioactive decay of the source. ∊ is the calibration factor for the TLD response (nC/cGy) measured with a Cs‐137 radiotherapy source with 250 Ci activity at the time of calibration. The Cs‐137 source was an old radiotherapy unit which is used for research and calibrations, not for treatment of patients. Two TLD chips were put on a 2 mm thick Plexiglas slab, and were exposed to a known amount of radiation. We used an ionization chamber detector to measure the total dose received by each set of chips. *E(r)* is a correction factor for the energy dependence of the TLD between the calibration beam and the Cs‐137 photon, which equals unity in this case. *d (T)* is a correction factor used to account for the decay of the source during irradiation. *d (T)* is assumed to be unity for Cs‐137 source with 30 y half‐life. *Flin* is the nonlinearity correction of the TLD response for the given dose. *Flin* was assumed 1.0 for the dose range of 10 to 100 cGy.

**Figure 2:**
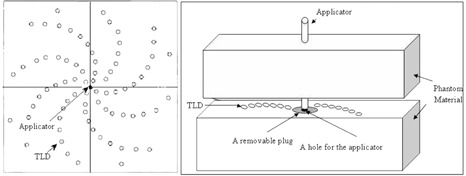
Schematic diagram of the experimental setup for measurement of the radial dose function.

**Figure 3:**
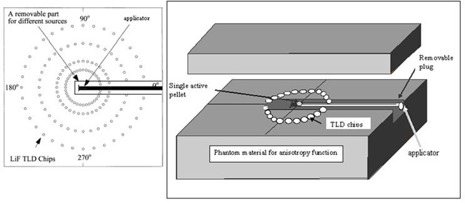
Schematic diagram of the experimental setup for measurement of anisotropy function (F(r, θ)).

Two $30\times 30\times 30\;{\text{cm}}^{3}$ Plexiglas phantoms were used to measure dose rate constant, radial dose function, and 2D anisotropy function of the source. Each phantom was composed of eight slabs with dimensions $30\times 30\times 3\;{\text{cm}}^{3}$, and two slabs with dimensions $30\times 30\times 2\;{\text{cm}}^{2}$, and two slabs with dimensions $30\times 30\times 1\;{\text{cm}}^{3}$. Central slab of both of the phantoms (slab with 1 cm thickness) were machined to accommodate TLD chips and the source holder. The experimental setup for measurement of radial dose function and anisotropy function is shown in Figs. 2 and 3. The position of the source holder was selected such that the center of the active pellets was at the plane of TLD chips.

### A.5 Dose rate constant

The dose rate constant value of a single spherical source in the first position inside the applicator was calculated as:

(3) $$\Lambda =\frac{\dot{D}(r_{0},\theta_{0})}{Sk}.$$

where $\overset{\cdot }{D}(r_{0},\theta_{0})$ is the dose rate at the reference point ($r_{0}=1\;\text{cm}$ and $\theta_{0}=90{}^{\circ}$), which was measured using LiF TLD in Plexiglas phantom and Monte Carlo calculations in water.

### A.6 Radial dose function

The radial dose function, $g_{x}(r)$ describes the attenuation in tissue of the photons emitted from the brachytherapy source. The radial dose function is defined as:

(4) $$g_{x}=\frac{\dot{D}(r,\theta_{0})}{\dot{D}(r_{0},\theta_{0})}\cdot \frac{G_{x}(r_{0},\theta_{0})}{G_{x}(r,\theta_{0})}$$

where $\overset{\cdot }{D}(r,\theta_{0})$ and $\left(r,\theta_{0}\right)$ and $\overset{\cdot }{D}(r_{0},\theta_{0})$ are the dose rates measured at distances of *r* and $r_{o}$, respectively, along the transverse axis of the source. $G_{x}(r,\theta )$ is known as the geometry function which takes into account the effect of the interior geometry of source on the dose distribution at a given point. The geometry function is defined by the AAPM TG‐43 as:

(5) $$\begin{array}{l} G_{p}(r,\theta )=r^{-2}\;\;\;\;\;po\text{int}-source\;\;\;\;\;\;approximation\\ G_{L}(r,\theta )=\{\begin{array}{l} \frac{\beta }{Lr\;\;\;\;\sin\theta }\;if\;\;\;\theta \neq 0{}^{\circ}\\ {\left(r^{2}-L^{2}/4\right)}^{-1}\;\;\;\;\;if\;\;\;\theta =0{}^{\circ} \end{array}Line-source\;\;\;\;approximation \end{array}$$

where β, in radians, is the angle subtended by the tips of the hypothetical line source with respect to the calculation point, P(r,θ)^(6)^ and calculated as:

(6) $$\begin{array}{l} \beta =\tan^{-1}((x+L/2)/y)-\tan^{-1}((x-L/2)/y)\\ x=r\cos\theta \;\;\;\;\;\;\;\;\;\;\;\;y=r\sin\theta \end{array}$$

### A.7 Anisotropy Function

Two‐dimensional anisotropy function, F(r,θ), is defined as:

(7) $$F(r,\theta )=\frac{\dot{D}(r,\theta )}{\dot{D}(r,\theta_{0})}\cdot \frac{G_{L}(r,\theta_{0})}{G_{L}(r,\theta )}$$

The values of anisotropy function were also obtained using TLD and MC simulations. The measurements were performed at distances of 1, 2, 3, 5, and 7 cm from the source center using a Plexiglas phantom that was machined to accommodate the TLD chips at different angles 30° to 165° relative to the source axis, in 15° increments. The measured values of dose rate at different positions were used for calculation of F(r, θ) according to Eq. (7). The anisotropy functions were also calculated in water using Monte Carlo simulation method. The simulations were performed at distances of 1, 2, 3, 4, 5, and 7 cm for different angles from the source center.

## III. RESULTS & DISCUSSION

### A.1 Dose rate constant

The measured dose rate constant of the Cs‐137 pellet source in position 1 inside the applicator, in Plexiglas phantom, was found to be $1.093\pm 0.08\;{\text{cGyh}}^{-1}U^{-1}$. The Monte Carlo calculated dose rate constant for a single pellet source in water phantom without the applicator was found to be $1.102\pm 0.06\;{\text{cGyh}}^{-1}U^{-1}$, and in presence of the applicator and non‐active pellets while the pellet in first position was active, was $1.095\pm 0.05\;{\text{cGyh}}^{-1}U^{-1}$. Pérez et al.^(4)^ have reported a value of 1.107 for dose rate constant of a single active pellet in water without the applicator. These results indicate a good agreement (less than 0.5%) between the present work and previous published data, including the 0.6% differences for the effect of the applicator and non‐active pellets.

### B.2 Radial dose function

**Figure 4:**
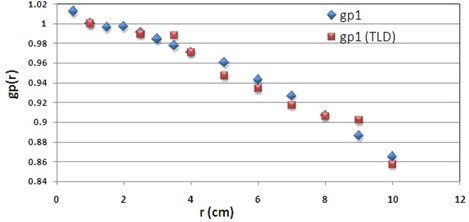
A comparison between measured and calculated radial dose function of the pellet.

The radial dose function for the pellet source for point source approximation can be obtained by Eq. (3). The Monte Carlo calculated values of $g^{p}(r)$ for the single pellet source without the applicator and in positions 1 to 6 inside the applicator are shown in Table 1. Figure 4 shows a comparison between measured and calculated radial dose function $\left(g^{p}1(r)\right)$ when the first pellet is active. These results indicate up to 3.3% smaller radial dose function of the source at 10 cm depth due to the presence of the applicator and non‐active pellets.

**Table 1:** Radial dose function for point source approximation for an active source without the applicator and a single active pellet in positions $1(g_{p}1)$ to $6(g_{p}6)$ inside the applicator.

*r (cm)*	$g_{P}$ *pellet source without the applicator*	$g_{p}1$	$g_{p}2$	$g_{p}3$	$g_{p}4$	$g_{p}5$	$g_{p}6$
0.5	1.007	1.012	1.013	1.013	1.012	1.011	1.012
1	1	1	1	1	1	1	1
1.5	0.994	0.996	0.993	0.995	0.995	0.995	0.995
2	0.998	0.997	0.992	0.989	0.989	0.992	0.990
2.5		0.991	0.984	0.983	0.983	0.986	0.984
3	0.985	0.984	0.974	0.979	0.979	0.978	0.981
3.5	0.979	0.977	0.968	0.980	0.979	0.972	0.979
4	0.968	0.971	0.958	0.971	0.970	0.964	0.971
5	0.956	0.960	0.953	0.948	0.949	0.953	0.948
6	0.943	0.943	0.939	0.943	0.939	0.939	0.938
7	0.927	0.926	0.923	0.924	0.919	0.922	0.917
8	0.911	0.907	0.894	0.909	0.905	0.899	0.905
9	0.893	0.886	0.886	0.873	0.876	0.883	0.878
10	0.870	0.865	0.858	0.842	0.850	0.860	0.851

### C.3 Anisotropy function

**Figure 5:**
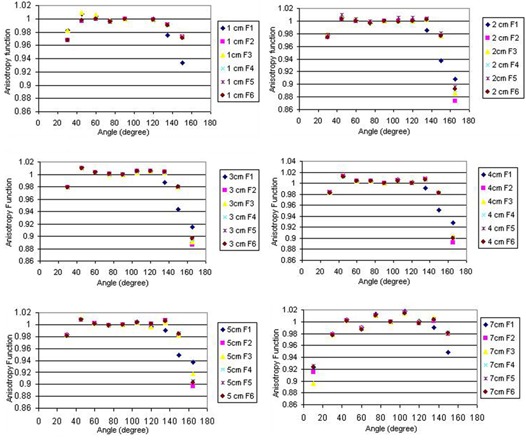
Anisotropy function of the active source in different positions inside the applicator at distances 1 to 7 cm.

**Figure 6:**
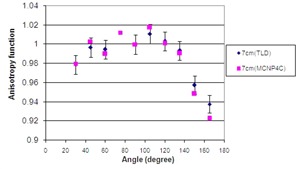
A comparison between measured and calculated values of F(r, θ) at distance 7 cm while the active source is in position 1 inside the applicator.

The Monte Carlo calculation of 2D anisotropy function for a single pellet source in different positions inside the applicator is shown in Fig. 5. The terms F1 to F6 used in Fig. 6 refer to the situations in which the active source is located in positions 1 to 6, respectively. The results indicate that the anisotropy around the source in positions 1 to 6 differ mostly for small angles relative to the longitudinal axis of the applicator, particularly along the applicator tip. The geometry of the applicator tip is the cause of such differences. The results of TL dosimetry for the source in position 1 are shown in Table 2. Figure 6 also compares the calculated and measured anisotropy function at distance 7 cm from the source center while the source in position 1 is inside the applicator. The results show excellent agreement between the measured and calculated values of anisotropy function of the source. According to the results, even at angles near 90°, there are always attenuation and scatter of the photons due to the presence of these components. Such dose reductions around a single pellet inside the applicator will be more pronounced for typical treatment procedures using more than one pellet (i.e., 24 or 36 pellets). Based on the American Brachytherapy Society recommendations for LDR brachytherapy for carcinoma of the cervix,^(16)^ the radiation therapy of vaginal cuff is performed based on the dose prescription normally to a point located at 5 mm depth from the applicator surface, approximately at mid‐portion of the active length. The applicator shielding would affect the dose of the prescription point in typical treatment procedures performed using several active pellets.

**Table 2:** Measured anisotropy function of the active source in different positions inside the applicator at distances 1 to 7 cm.

	*1 cm*	*2 cm*	*3 cm*	*5 cm*	*7 cm*
15°		0.892	0.903	0.921	0.920
30°			0.956	0.961	0.979
45°	0.973	0.998	1	0.996	0.997
60°	0.989		0.999	0.992	0.995
75°		0.997	1.003	1.005
90°	1	1	1	1	1
105°		1.002	1.010	1.007	1.010
120°			1.003	1.006	1.003
135°	0.894	0.936	0.948	0.988	0.993
150°			0.944	0.953	0.958
165°		0.899	0.902	0.939	0.938

In addition, the real target can be a point located along the longitudinal axis of the source; therefore, the attenuation affects the target point more than the prescription point. Hence, the attenuation due to the applicator and pellets has more effect on target point than prescription point. Such reduction in dose around the vaginal applicator is expected to be observed in cases where tandem and ovoids are used in gynecological brachytherapy. The larger number of active pellets used in tandem‐ovoid sets and the more complicated geometry of such cases would cause even more errors in TPS dose calculation than the cases that use vaginal applicator. Williamson et al.^(17)^ showed that for typical clinical applications, not accounting for the shielding effects of applicators would overestimate the dose at ICRU38 rectum and bladder reference points by 12%–25%. Many other factors are important in the outcome of brachytherapy and should be considered in addition to the applicator shielding effect such as the initial volume of disease, the ability to displace the bladder and rectum, the degree of tumor regression during pelvic irradiation, and institutional practice and the duration of treatment (according to the suggestions of ABS, it should be less than eight weeks).^(16)^

The high‐tech environment of HDR brachytherapy makes it an acceptable alternative to LDR, which is why LDR remote afterloading units are not commonly used in US. The main reasons that HDR brachytherapy has become more popular in clinical practices are its ability to treat on an outpatient basis and the fact that it takes less time to treat patients.^(18)^ However, anyone considering changing from LDR to HDR afterloading therapy should be aware that we are not yet certain about the biological impact of HDR brachytherapy.^(^ ^16^ ^,^ ^19^ ^)^ According to Task Group #59 of AAPM (TG‐59), normal tissue toxicity is expected to be more in HDR treatment than LDR.^(18)^ The complicated treatment system of HDR brachytherapy requires more expert physicists, dosimetrists, and radiation therapists, as each error may have severe consequences. Finally, the failure of HDR sources to retract would result in very high radiation dose to patients

and unit operators. Such disadvantages of HDR brachytherapy ensures that the clinical use of LDR will still be used in gynecological brachytherapy. It should be noted that presently in North America, the majority of brachytherapy remote afterloading units are HDR units (325 out of 500 units sold are HDR units).^(18)^ In other countries, especially in developing countries, LDR brachytherapy is still widely used. For instance in Iran, LDR gynecological brachytherapy is still the method of choice for treatment of patients.

## IV. CONCLUSIONS

The TG‐43 dosimetric parameters of the model Selectron Cs‐137 LDR pellet source were studied using standard methods employing thermoluminescent dosimeters in water‐equivalent phantoms, and MCNP4C Monte Carlo code. According to the results, dose rate constant of the Cs‐137 model Selectron source is comparable to those of commercially available sources. Moreover, the anisotropy functions of this source, in position 1 are in excellent agreement with Monte Carlo calculated data. According to this study, the position of the single active source inside the applicator doesn't have any significant effect on radial dose function of the source. The effect of the position of the active source has a more significant effect on F(r, θ) along the applicator tip; this effect is due to the geometry of the applicator tip. Using the anisotropy functions in the presence of the applicator and inactive pellets, the dose distribution around different compositions of active and inactive pellets used in treatment of vaginal cancer can be assessed more precisely than commercial treatment planning systems.

In summary, the attenuation of the applicator wall and adjacent active and inactive pellets in the source reduces the dose distribution of the Cs‐137. This effect is larger at smaller angles relative to the longitudinal axis of the applicator.

## ACKNOWLEDGMENTS

The authors wish to acknowledge the personnel in the Radiotherapy Department of Namazi Hospital. We would also like to thank the Radiation Research Center of Shiraz University.
